# Cardiovascular Disease Risk Stratification Using Hybrid Deep Learning Paradigm: First of Its Kind on Canadian Trial Data

**DOI:** 10.3390/diagnostics14171894

**Published:** 2024-08-28

**Authors:** Mrinalini Bhagawati, Sudip Paul, Laura Mantella, Amer M. Johri, Siddharth Gupta, John R. Laird, Inder M. Singh, Narendra N. Khanna, Mustafa Al-Maini, Esma R. Isenovic, Ekta Tiwari, Rajesh Singh, Andrew Nicolaides, Luca Saba, Vinod Anand, Jasjit S. Suri

**Affiliations:** 1 Department of Biomedical Engineering, North-Eastern Hill University, Shillong 793022, India; bhagawatimrinalini07@gmail.com (M.B.); sudip.paul.bhu@gmail.com (S.P.); 2 Division of Cardiology, Department of Medicine, University of Toronto, Toronto, ON M5S 1A1, Canada; lauraevemantella@gmail.com; 3 Division of Cardiology, Department of Medicine, Queen’s University, Kingston, ON K7L 3N6, Canada; amerjohri@gmail.com; 4 Department of Computer Science and Engineering, Bharati Vidyapeeth’s College of Engineering, New Delhi 110063, India; siddgupta462@gmail.com; 5 Heart and Vascular Institute, Adventist Health St. Helena, St. Helena, CA 94574, USA; lairdjr@ah.org; 6 Stroke Diagnostic and Monitoring Division, AtheroPoint™, Roseville, CA 95661, USA; drindersingh1@gmail.com (I.M.S.); vinodanand2010@gmail.com (V.A.); 7 Cardiology Department, Apollo Hospitals, New Delhi 110076, India; drnnkhanna@gmail.com; 8 Allergy, Clinical Immunology and Rheumatology Institute, Toronto, ON M5G 1N8, Canada; almaini@hotmail.com; 9 Department of Radiobiology and Molecular Genetics, National Institute of The Republic of Serbia, University of Belgrade, 11001 Belgrade, Serbia; isenovic@yahoo.com; 10 Department of Computer Science, Visvesvaraya National Institute of Technology (VNIT), Nagpur 440010, India; ekta.tiwari.ai@gmail.com; 11 Division of Research and Innovation, UTI, Uttaranchal University, Dehradun 248007, India; director.ri@uumail.in; 12 Vascular Screening and Diagnostic Centre, University of Nicosia, Nicosia 2417, Cyprus; anicolaides1@gmail.com; 13 Department of Radiology, Azienda Ospedaliero Universitaria, 40138 Cagliari, Italy; lucasabamd@gmail.com; 14 Department of CE, Graphic Era Deemed to be University, Dehradun 248002, India; 15 Department of ECE, Idaho State University, Pocatello, ID 83209, USA; 16 University Center for Research & Development, Chandigarh University, Mohali 140413, India; 17 Symbiosis Institute of Technology, Nagpur Campus, Symbiosis International (Deemed University), Pune 412115, India

**Keywords:** cardiovascular disease risk, machine learning feature extraction, hybrid deep learning, scientific validation, performance evaluation, reliability, and stability

## Abstract

Background: The risk of cardiovascular disease (CVD) has traditionally been predicted via the assessment of carotid plaques. In the proposed study, AtheroEdge™ 3.0_HDL_ (AtheroPoint™, Roseville, CA, USA) was designed to demonstrate how well the features obtained from carotid plaques determine the risk of CVD. We hypothesize that hybrid deep learning (HDL) will outperform unidirectional deep learning, bidirectional deep learning, and machine learning (ML) paradigms. Methodology: 500 people who had undergone targeted carotid B-mode ultrasonography and coronary angiography were included in the proposed study. ML feature selection was carried out using three different methods, namely principal component analysis (PCA) pooling, the chi-square test (CST), and the random forest regression (RFR) test. The unidirectional and bidirectional deep learning models were trained, and then six types of novel HDL-based models were designed for CVD risk stratification. The AtheroEdge™ 3.0_HDL_ was scientifically validated using *seen* and *unseen* datasets while the reliability and statistical tests were conducted using CST along with *p*-value significance. The performance of AtheroEdge™ 3.0_HDL_ was evaluated by measuring the *p*-value and area-under-the-curve for both *seen* and *unseen* data. Results: The HDL system showed an improvement of 30.20% (0.954 vs. 0.702) over the ML system using the *seen* datasets. The ML feature extraction analysis showed 70% of common features among all three methods. The generalization of AtheroEdge™ 3.0_HDL_ showed less than 1% (*p*-value < 0.001) difference between *seen* and *unseen* data, complying with regulatory standards. Conclusions: The hypothesis for AtheroEdge™ 3.0_HDL_ was scientifically validated, and the model was tested for reliability and stability and is further adaptable clinically.

## 1. Introduction

Globally, the primary cause of death is cardiovascular disease (CVD) [1]. Comorbid conditions such as diabetes, hypertension, and a sedentary lifestyle contribute to the rise in CVD mortality [2,3]. Therefore, timely and non-invasive CVD risk stratification is desperately needed. A useful screening technique for the identification of cardiovascular disease and cardiovascular events (CVE) is the use of carotid ultrasonography as a surrogate biomarker [4,5]. This non-invasive diagnostic tool is highly effective in evaluating atherosclerotic plaque [6,7,8]. The primary indicators obtained from carotid artery ultrasound scans are carotid intima-media thickness (cIMT) [9,10,11], total plaque area (TPA) [11,12,13,14], and maximum plaque height (MPH) [15].

It has recently been demonstrated that a significant and trustworthy indicator for CVE and CVD is intraplaque neovascularization (IPN), an indicator of the instability in plaque and its development [16]. The CAD and carotid ultrasound-based image phenotype (CUSIP) connection were previously studied using standard regression algorithms, most of which were linear in form [17,18]. When addressing relationships of non-linear type among the risk factors and CAD ground truth, it was therefore an oversimplification. Regression-based methods also have the drawback of being unable to manage large and varied cohort sizes. These CVD risk models cannot, therefore, reliably forecast the occurrence of CVD events. Therefore, more sophisticated instruments that can precisely calibrate the CVD risk assessment are required [19,20].

Artificial intelligence (AI)-based methods have recently been demonstrated to outperform conventional statistical-based models [21]. AI has shown promise in several medical imaging applications, including radiology [21,22,23,24,25], dermatology [26,27,28,29], ophthalmology [30,31,32,33,34,35], cardiology [36,37,38,39], endocrinology [40,41,42,43], and, more recently, carotid ultrasonography [44,45,46] for the prediction of CVD risk. These AI systems can generate trained models in offline mode to understand the link between the training risk variables with the CVD outcomes [47]. To establish the events of CVD in an online form, the models that are trained are subsequently utilized to modify the test risk variables [27,48].

The field of medical imaging has recently been dominated by deep learning (DL) [49,50,51]. DL models are integrated with architectures having multiple layers for quick feature selection and have the added benefits of automated feature extractions [52,53]. Information from data sequences having both forward and backward temporal dependency is well captured via bidirectional models. Bidirectional systems are more robust than unidirectional ones because each element of an input sequence obtains signals from two main sources, namely the past and the present [54]. Additionally, by merging the unidirectional and bidirectional DL systems for the HDL architectural design, this study has produced sophisticated protocols.

**Figure 1:**
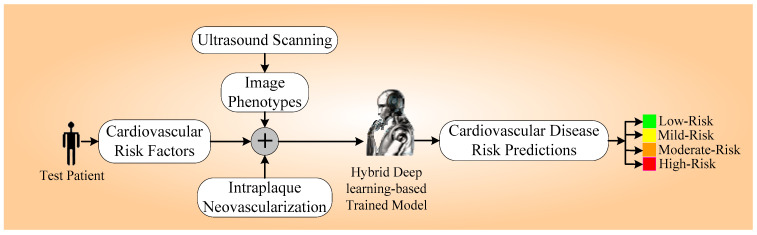
AtheroEdge™ 3.0_HDL_ online HDL-based system for prediction of CVD.

Figure 1 displays the novel online system for the prediction of CVD risk, called AtheroEdge 3.0HDL. The system displays the combination of IPN, picture phenotypes, and cardiovascular risk factors. This combination is fed into the trained system for the prediction of CVD risk, generating a risk prediction in a multi-risk granular way (shown in four colors). Three methods were used for the feature selection (not depicted in the figure): principal component analysis (PCA) pooling, the chi-square test (CST), and random forest regressor (RFR) for the machine learning system.

The AtheroEdge™ 3.0_HDL_ system undergoes performance evaluation via plotting to generate operating characteristics (ROCs) along with their *p*-value. The scientific validation of the AtheroEdge™ 3.0_HDL_ system was performed by using unseen data, and the reliability was checked using the statistical tests. We further benchmark the HDL-based models against unidirectional and bidirectional DL models along with the ML models (three) which are already present, namely random forest (RF) [55], gradient boost (GB) [56], and support vector machine (SVM) [56,57].

The proposed study is organized as follows: Section 2 discusses the background literature, and Section 3 contains the methodology. The results are shown in Section 4, and the performance evaluation is shown in Section 5. The statistical tests and reliability analysis are presented in Section 6. Section 7 covers the crucial discussions. Lastly, Section 8 presents conclusions.

## 2. The Background Literature

The evolution of CVD risk prediction systems has seen significant advancements over the years, with the integration of various methodologies. The journey started with the traditional systems between the 1980s and 1990s. Initial CVD risk prediction systems were based on traditional risk variables such as gender, age, cholesterol levels, blood pressure, and smoking status. The work by Wilson et al. [58] (1998) laid the groundwork for risk stratification in CVD and influenced subsequent studies and guidelines. The risk factor categories and the risk prediction model developed in this study have been instrumental in shaping preventive strategies and interventions for coronary heart disease (CHD).

The next phase was the inclusion of multivariate risk assessment (2000s). The focus shifted to multivariable risk assessment models that considered a broader range of factors, including diabetes, family history, and body mass index (BMI) (see Conroy et al. [59]). The systematic coronary risk evaluation (SCORE) project significantly contributed to CVD assessment in European populations. Its emphasis on region-specific models and the development of risk charts has influenced clinical practice and guidelines, providing a standardized method for evaluating CVD risk in different European contexts.

The next is the emergence of ML systems (2010), namely, RF, SVM, and neural networks, which were introduced to enhance predictive accuracy by analyzing large datasets with diverse variables. Goff Jr. et al. [60] (2014) showed that in comparison to previous risk calculators, the American College of Cardiology (ACC)/American Heart Association (AHA) calculator included a broader set of risk factors, aiming for a more comprehensive assessment of CVD. The risk calculator placed a significant emphasis on the role of statin therapy in primary prevention, recommending statin therapy for individuals with a 10-year ASCVD risk exceeding a certain threshold.

The incorporation of the image data was another advancement that included imaging phenotypes, namely cIMT (so-called, CUSIP), TPA, and coronary artery calcium scores, into risk prediction models for improved accuracy. Several scientific groups have provided examples of how to employ cIMT and TPA for CVD risk assessment [5]. AtheroEdge^TM^ 1.0 was designed which took advantage of CUSIP measurements leading to the CVD risk assessment. Subsequently, the 10-year risk prediction and CVD risk classification features of office-based biomarkers (OBBM), laboratory-based biomarkers (LBBM), and CUSIP were incorporated into the architecture of the AtheroEdgeTM 2.0 system. With the AtheroEdgeTM technology, scale-space image processing techniques were used for automated lumen-intima (LI) and media-adventitia (MA) border recognition for the far wall of the carotid arteries. There were several applications made available [61].

Detrano et al. [62] (2008) found a significant association between the degree to which coronary artery calcium (CAC) is present and the risk of future coronary events across all four racial or ethnic groups. The CAC scores were identified as a strong and independent predictor of coronary events, providing additional information beyond conventional risk factors. While the assessed value of CAC was consistent across the groups, some differences were observed in the CAC scores’ distribution among the different racial or ethnic groups. The study highlighted the utility of CAC scoring, measured using CT scans, as a valuable tool for predicting CVE in a diverse population. This information could be valuable for risk stratification and guiding preventive interventions.

The latest and ongoing phase is DL and electronic health records (2015-present). Utilization of DL techniques includes Convolutional Neural Networks (CNNs) and recurrent neural networks (RNN), to extract patterns from electronic health records (EHRs) and improve risk prediction accuracy. Poplin et al. [63] (2018) demonstrated the feasibility of leveraging DL algorithms to extract valuable information from retinal fundus photographs for predicting cardiovascular risk factors. This innovative approach could open avenues for non-traditional methods of risk assessment in cardiovascular medicine.

## 3. Methodology

The role of HDL design for CVD risk stratification is the main objective. To accomplish this, we utilize the base DL models, namely unidirectional DL and bidirectional DL models. These are then combined in a tandem fashion to generate the fused HDL models. While the novel architectures are designed for CVD/stroke risk stratification, they need to be optimized via automated feature selection in the HDL system.

This section is therefore divided into the following seven subsections: The patient demographics and baseline characteristics are presented in Section 3.1. The acquisition of ultrasound images is shown in Section 3.2. The gold standard and CVD endpoint such as angiographic score is discussed in Section 3.3. The overall architecture is shown in Section 3.4, while the experimental protocol is presented in Section 3.5. The loss function adopted is shown in Section 3.6 and, lastly, Section 3.7 shows the performance metrics for AtheroEdge™ 3.0_HDL_.

### 3.1. Patient Demographics and Baseline Characteristics

In total, 500 patients present in our cohort were divided into four classes based on their coronary angiography number (CAS): class 0 represented no risk or very little risk, class 1 represented mild risk, class 2 represented a moderate form of risk, and lastly, class 3 represented high risk. A total of 160 patients had acute coronary syndrome (ACS), 13 had unstable angina (UA), and 114 patients had stable angina. Moreover, 139 of the 500 patients received stents. Every subject had 39 risk variables which are further grouped into four subgroups: (i) the OBBM cluster comprising 17 covariates; (ii) the LBBM cluster with seven risk factors; (iii) the CUSIP cluster with three variables, so-called radiomics-based biomarkers; and (iv) MedUse. The OBBM cluster had the following mean scores: age (64.49 years), gender (349, 69.8%), obesity (215, 43.0%), ethnicity (486, 97.2%), BMI (31.12 kg/m^2^), hypertension (338, 67.6%), angina (124, 24.8%), diastolic blood pressure (76.7 mmHg), systolic blood pressure (135.35 mmHg), smoking history (330, 66%), casual-smoker (15, 3%), current-smoker (100, 20%), previous-smoker (218, 43.6%), drinks (4.94 ± 10.4 per week), family history of diabetics (195, 39.0%), premature CVD in the family (146, 29.2%), and CVD in the family (321, 64.2%). The LBBM cluster had the following risk factors with their respective means in parentheses: creatinine (83.99 ± 22.6 μmol/L), pre-diabetic (20, 40%), hyperlipidemia (288, 57.6%), type II diabetes (114, 22.8%), type I diabetes (5, 1.0%), estimated glomerular filtration rate (78.96 mL/min/1.73 m2), and diabetes of any type (1188, 23.6%). The CUSIP subgroup has the following means: TPA (47.68 mm^2^), MPH (2.64 mm), and IPN (1.16). The MedUSE group had the following means: angiotensin-converting enzyme (ACE) inhibitors (191, 38.2%), HMG-Co reductase inhibitors (272, 54.4%), angiotensin receptor blockers (ARBs) (45, 9.0%)), other antilipemic agents (9, 1.8%), alpha-blockers (30, 6.0%), calcium channel-blockers (93, 18.6%), beta-blockers (236, 47.2%), diuretics (99, 19.8%)), anti-platelet medications (368, 73.6%), insulin (38, 7.6%), anti-anginals and NSAIDS (81, 16.2%)), and non-insulin diabetes medications (72 (14.4%)) (see Appendix A, Table A1).

### 3.2. Acquisition of Ultrasound Scans and Intraplaque Neovascularization

Automated angiography screening has been popular for obtaining ultrasound scans [64,65]. It has been well proven that carotid artery disease is the surrogate biomarker for coronary artery disease [4]. Further, ultrasound imaging for the carotid artery is non-invasive and ergonomic [66,67]. All patients are made to undergo B-mode focused carotid ultrasonography imaging by using a Vivid E9 from GE Healthcare. The system has an array of 9L-D linear transducers of 2.4–10 MHz. As shown in the other studies from our group, the collection of two image phenotypes, namely TPA and MPH, was computed using longitudinal ultrasound scans using the guidelines of the American Society of Echocardiography (ASE). The MPH is the separation of the media-adventitia (MA) and the lumen-intima (LI), considering both sides of the neck. When calculating the TPA, which is the total amount of plaque present in the carotid arteries internally and externally, located 10 mm (1 cm) closest to the bifurcation of the carotid artery. According to the standard definition, when the focal structure encroaches into the lumen zone and is more than 1.5 mm in diameter or 50% of the cIMT, it is classified as a carotid plaque.

The ultrasound imaging (contrast-based) of carotid artery plaque is used by the authors to quantify IPN for the detection of plaque instability and progression. IPN was graded 0, 1, 2, and 3 as per the contrasting microbubbles migration from the adventitial wall to the plaque core. These classifications are as follows: the numbers 0 and 1 indicate that there are no microbubbles visible, and 2 and 3 indicate that the plaque region is partially filled or filled with microbubbles. The total score was calculated by averaging the IPN grades on the sides of the neck. The resulting values were useful and efficient metrics for CVE and CAD.

### 3.3. Cardiovascular Disease Endpoint: Angiographic Score

Angiograms, graded by investigators who were blind to the clinical variables of the subjects, serve as surrogate biomarkers for CVD events. As we mentioned in our earlier work, we employ the GE Healthcare Vivid platform 2000, for the Standard Judkins ultrasound examination procedure. The group of expert cardiologists objectively marked the obtained angiograms from the coronary artery. The left anterior descending (LAD), left main, circumflex, and right coronary arteries (RCA) are the places from where the arterial stenosis grades were obtained. A disease was classified as minor if its stenosis was less than 19% in any segment, mild if it was between 20 and 49%, moderate if it was between 50 and 69%, and severe if it was greater than 70% in any segment and less than 50% in the left main coronary artery (LCA). While left ventricular echocardiography pictures can be used to automatically classify patients with CAD, coronary artery angiography is still the ground truth for CVD screening and CAD assessment. The use of a multiclass ground truth for CVD risk prediction has been demonstrated in a study employing the AtheroEdge™ 3.0_HDL_ system [54].

### 3.4. Overall Architecture

The architecture for AtheroEdge™ 3.0_HDL_ was designed with several DL architectures and protocols. The data are separated into subsets, namely training and testing data, where the training data are used for training the DL algorithm while testing data are used for the prediction of CVD risk. One of the key components to consider is data preparation and selecting the correct cross-validation methodology to help validate the robustness of the AI algorithm. This is accomplished by systematically partitioning the data into various non-overlapping training and testing subsets. This maximizes coverage of the learning model over all participants.

#### 3.4.1. Data Preparation and Pre-Processing

**Figure 2:**
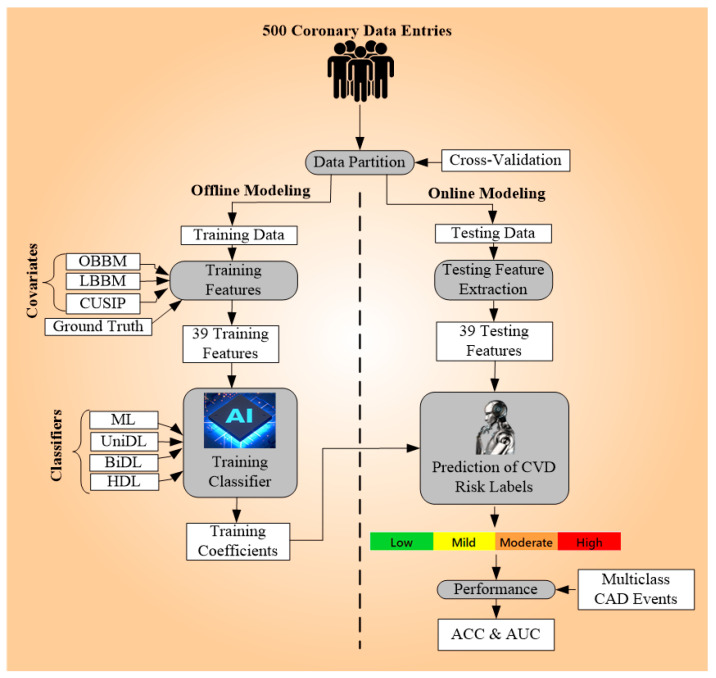
Overall architecture of AtheroEdge™ 3.0_HDL_.

In light of this, we divided the AtheroEdge™ 3.0_HDL_ general architecture into four main parts. The first component is for data pre-processing, which works in tandem with the second component, for data partitioning. Component three generates training models offline (see Figure 2), while component four estimates the risk of CAD or CVD using the testing datasets. Three primary procedures are involved in data preparation: (i) normalizing the data using a conventional scalar platform that places the features on a scale from 0 to 1 [68] and (ii) choosing the dominating features using three paradigms, namely PCA, CST, and RFR. The data are augmented using the synthetic minority over-sampling technique (SMOTE) [69,70,71,72,73] to overcome the problems of overfitting due to the small data size. Additionally, we used random sampling to further increase the data size to 5000 entries.

The data were preprocessed before being split into training and testing sets. Using the Sklearn library in Phyton, the quality control procedure involves applying normalization, scaling, min–max, and scaler. Each feature is normalized using the MinMaxScaler() method, which scales the data to a range. With a default of 0 and 1, the MinMaxScaler() function scales each feature separately to give the values a specified minimum and maximum value [74,75]. Unlike the self-capable DL system, the procedures for feature extraction and choosing features were solely implemented in the ML system.

#### 3.4.2. Model Building

The third architectural component is a model-building process that uses HDL classifiers to produce the offline coefficients. Risk factors and other inputs are used to feed these classifiers. The fourth and last component is a prediction paradigm that modifies the test data to forecast the CVD risk using a trained model. The employed prototype will also predict the projected CVD risk for every ten combinations in a cyclic sequence, guaranteeing that there is no repetition of training data in the test data and that all combinations are mutually exclusive. The performance evaluation component is computed given the ground truth scores and the predicted CVD risk using the online system. In this component, we compute the AUC using ROC analysis.

#### 3.4.3. The Rationale behind Using RNN, GRU, and LSTM

Atherosclerosis is a condition that affects people of many ethnicities and is characterized by the buildup of lipids, cholesterol, and plaque on the artery walls, which block the arteries. This study’s dataset consists of a group of individuals with coronary artery disease (CAD) who are older, of the same ethnicity (usually Caucasian), and share one or more of the other three criteria. The cases under consideration share many similarities, with very slight variations. These patients’ medical conditions determine which risk groups they are exposed to. We may consequently rank the patients in ascending order of coronary artery disease risk because they are all drawn from the same distribution of statistics and show up for health exams in a prearranged window of time. Mindfully, the main premise is that all subjects are relatively similar in all other aspects; it can be assumed that this new distribution represents atherosclerosis progression in the periods of discrete time for each subject depending on the risk at different phases of the disorder [54].

Sequence models, such as RNN, are among the most well-known deep learning models for their proficiency at extracting features in a temporal manner and semantics at a high-level from discrete and sequential data. This is the main rationale for classifying the risk of developing atherosclerosis using RNN and contrasting it with conventional ML-based classification systems. For such representations, LSTM, RNN, and GRU are the best options [76,77,78,79].

### 3.5. Experimental Protocols

To validate the hypothesis, we have created three sets of protocols. Each of the experimental protocols follows the cross-validation approach, in which the data are divided into two parts: training and testing. Four sets of cross-validation paradigms are implemented, namely two-fold (K2), four-fold (K4), five-fold (K5), and ten-fold (K10), where K2, K4, K5, and K10 constitute the following partition ratios: 50:50, 75:25, 80:20, and 90:10. Each of the cross-validation protocols is implemented in three kinds of experimental protocols, namely experimental protocol 1 (EP1), experimental protocol 2 (EP2), and experimental protocol 3 (EP3).

#### 3.5.1. Experimental Protocol 1: Three Unidirectional Models

**Figure A1:**
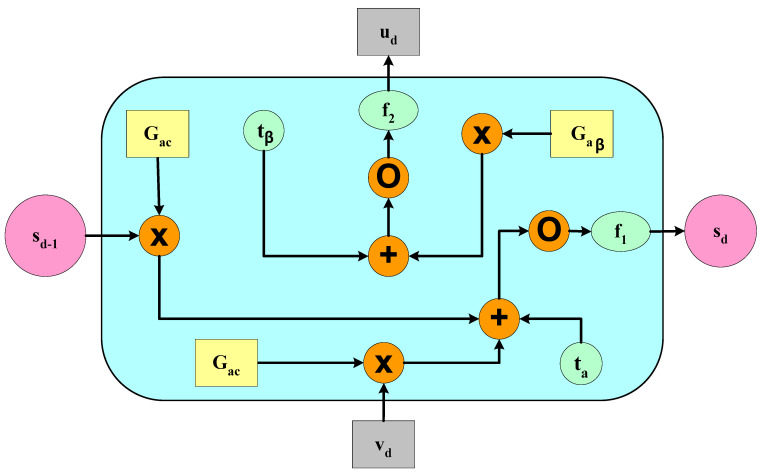
RNN Architecture.

**Figure A2:**
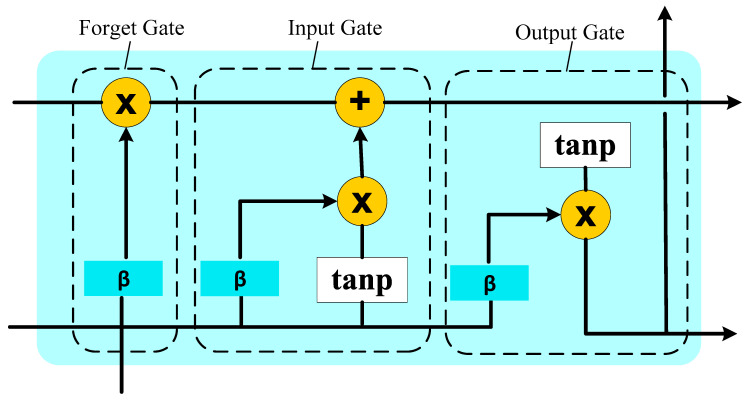
LSTM Architecture.

**Figure A3:**
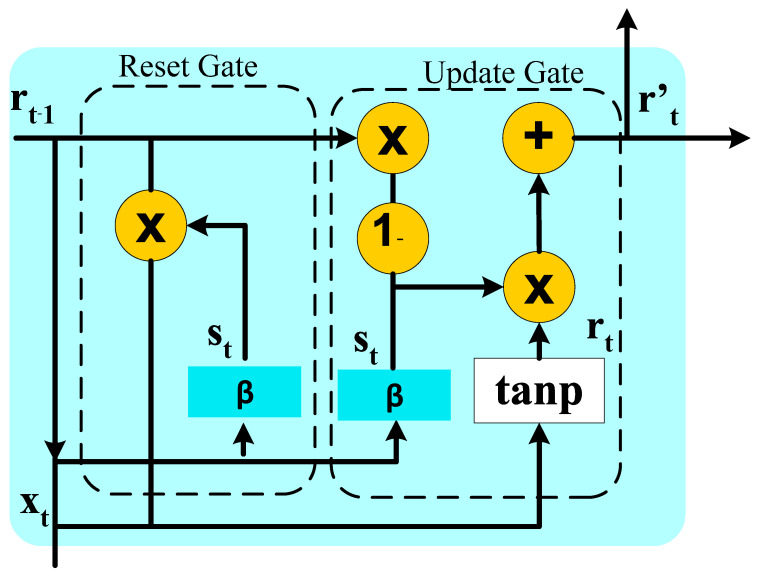
GRU Architecture.

The unidirectional DL models RNN [80], gated-recurrent units (GRU) [81,82], and long short-term memory (LSTM) [83] have all been taken into consideration by the authors in EP1. RNNs work particularly well in sequence-based applications such as series-of-time prediction, audio recognition, and processing of natural language. However, ordinary RNNs struggle to capture long-term correlations due to issues like bursting or fading gradients (see Appendix B, Figure A1). To address the vanishing gradient problem, one type of RNN architecture known as LSTM (see Appendix B, Figure A2) was developed. Its memory cells and various information-flow regulating gates make it more efficient at determining distant links within sequences. GRU is another RNN variant that is similar to LSTM but has a marginally different topology. It has been proven to perform well in a variety of sequence-based tasks and is outfitted with gating devices to control the flow of information (see Appendix B, Figure A3). These models are utilized for K2, K4, K5, and K10, which are the various CV regimens.

#### 3.5.2. Experimental Protocol 2: Three Bidirectional Models

**Figure A4:**
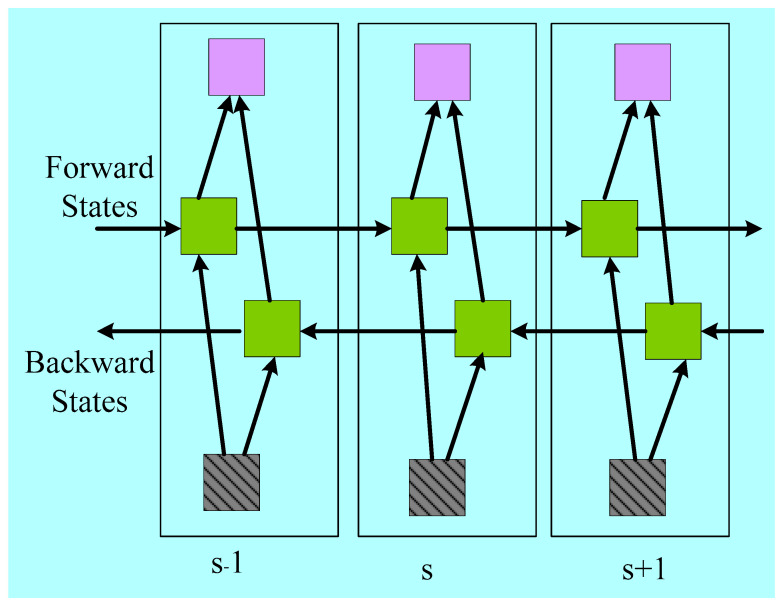
BiRNN Architecture.

**Figure A5:**
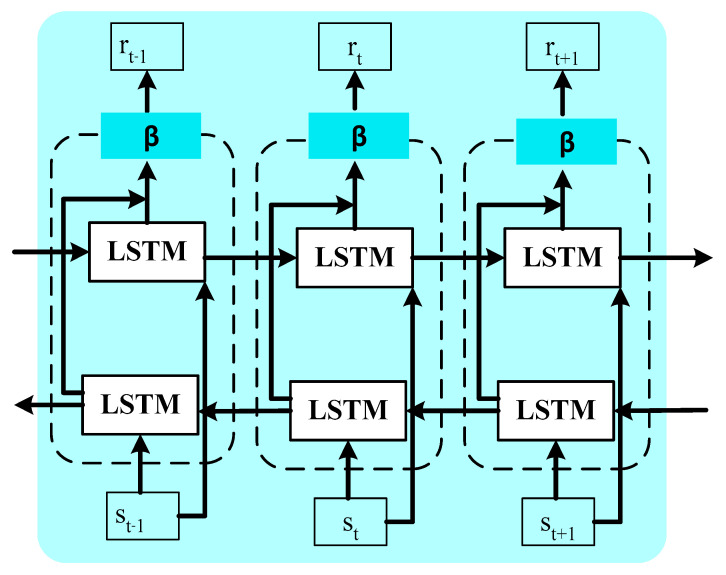
BiLSTM Architecture.

**Figure A6:**
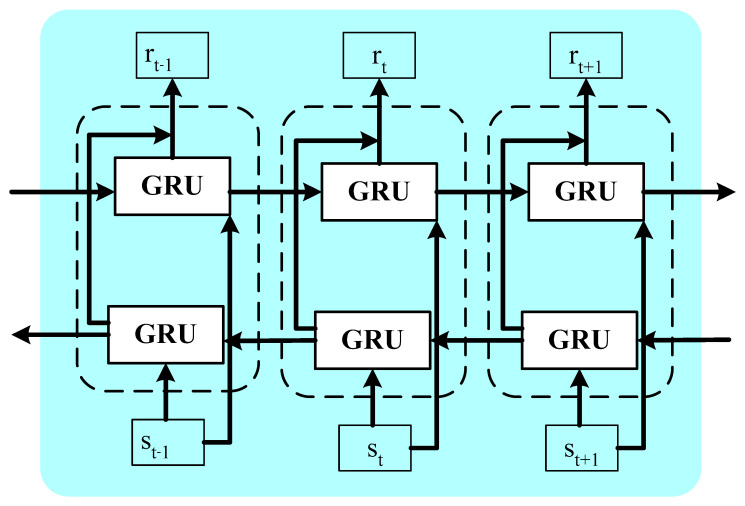
BiGRU Architecture.

The EP2 had the bidirectional DL systems, namely BiRNN [84] (See Appendix C, Figure A4), BiLSTM [85] (See Appendix C, Figure A5), and BiGRU [77,86] (See Appendix C, Figure A6).

##### BiRNN

In order to analyze sequential data, the input sequence traverses in two directions, namely, forward and backward, at same the time in BiRNN. The network performs a forward pass to gather data from the past and a backward pass for gathering data from the future for each time step. Therefore, the representation of the context present in each time step is holistically accomplished via the combination of the hidden states from both sides. Due to the bidirectional manner of operation, BiRNNs are mainly beneficial for applications where deep context comprehension is required, such as time-series analysis, recognition of voices, processing natural languages, CVD risk prediction, and depressive illness prediction. The network can more efficiently recognize dependencies and connections that are present in the sequential data. By using backpropagation to adjust the parameters of the network during training, the accuracy of the predictions or classifications the network may make based on the bidirectional contextual input is maximized.

##### BiLSTM

The BiLSTM model is based on the bidirectional flow for understanding the sequential input. It records the information in a contextual form from both the past and future by comparing the forward and backward layers of the LSTM model. By managing input, forget, and output gates, the LSTM units govern the flow of information and preserve cells of memory for storing relevant data at each time step. The neural network may comprehend long-term relationships and linkages along the whole sequence as they are of bidirectional nature. Concatenating the hidden states that are present on both sides generally results in the representation of the context more completely. This architecture is particularly useful for issues like time series, natural language processing, and illness prediction where accurate prediction necessitates knowledge of both preceding and subsequent components. Back-propagation changes the parameters of the network during training over time, which improves the capacity for representing the sequential relationship.

##### BiGRU

BiGRU is based on two parallel layers, namely forward and backward layers, for assessing the sequential input. Every time step, data are continuously collected by the forward GRU from earlier items and by the reverse GRU from later elements. Then, to create a complete picture of the context, the concealed states on both ends are frequently combined. BiGRU networks are useful for characterizing linkages and interactions within sequential data since they are bidirectional. Throughout the training, backpropagation processes gradually update the network’s properties, optimizing the capacity of the network to generate accurate classifications based on reciprocal contextual input.

#### 3.5.3. Experimental Protocol 3: Six Hybrid Models

**Figure A7:**
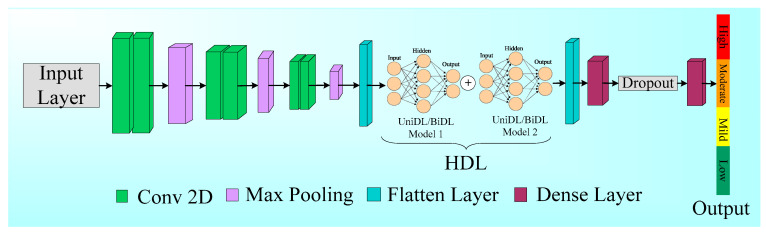
Hybrid deep learning architecture; UniDL: unidirectional DL; BiDL: bidirectional DL.

EP3 features a hybrid architecture which has the combination of the unidirectional DL and bidirectional DL systems. We design a combination of unidirectional DL or bidirectional DL models. In combination A, we combine both the models to create a unidirectional DL model. In combination B, we have combined both models to be bidirectional DL models. In combination A, we have three combinations, namely RNN + GRU, RNN + LSTM, and LSTM + GRU. In combination B, there are three combinations, namely: BiRNN + BiGRU, BiRNN + BiLSTM, and BiLSTM + BiGRU (See Appendix D, Figure A7). The HDL models were created by connecting the unidirectional and bidirectional models in tandem to obtain a better performance when compared to other models.

### 3.6. Loss Function and Training Parameters

The categorical cross entropy (CCE) loss function [87,88,89] is used for the calculation of the loss during the training of the system. Further, the losses obtained during the training and validation of the system were plotted against the number of epochs (100) present in the system. This type of loss function is used for the balanced distribution of data. The CCE loss function was selected over the other types as it is best suited for the binary variables. All other loss function types can be further added as future work. The binary cross entropy loss has been described in the equation below:

£_CCE_ = −(g log (p) + (1 − g) log (1 − p) (1)

where £_CCE_ is the binary cross-entropy loss, g represents the true label of the ith instance (either 0 or 1 for binary classification), and p represents the predicted probability that the ith instance belongs to class 1. Additionally, the training parameter used is as follows: the optimizer used is Adam [90], the number of epochs is 100, the batch size is 32, and the learning rate is 0.001. We utilized the early stopping method to reduce overfitting. Using the specified parameters, we trained all 12 models and their combinations with the 4 different cross-validation protocols. Afterward, we determined the most optimal model by comparing their performance metrics and selecting the best model based on this comparison.

### 3.7. Performance Metrics

The following phrases are utilized to derive the performance metrics: The number of cases that the model correctly classifies as positive is known as the True Positive (TP) value. The number of cases that the model correctly classifies as negative is known as the True Negative (TN) value. The number of times a negative instance is mistakenly classified as positive via the model is known as the False Positive (FP) value. The number of occurrences that the model mistakenly classifies as negative when they are positive is known as the False Negative (FN) value.

The percentage of accurate forecasts among all the predictions the model makes is known as accuracy. The percentage of accurate positive forecasts among all real positive occurrences is known as sensitivity. The performance indicator known as specificity counts the percentage of false positives that the model misidentified out of all the real negative cases. For varying classification thresholds, the receiver operating characteristic (ROC) curve represents the trade-offs between the TPR and FPR. By computing the area under the ROC curve, the area under the curve (AUC) measures the overall quality of the model.

(2) $$Accuracy\left(ACC\right)=\frac{\left(TP+TN\right)}{\left(TP+FP+FN+TN\right)}$$

(3) $$Sensitivity\left(SEN\right)=\frac{TP}{\left(TP+FN\right)}$$

(4) $$Specificity\left(SPEC\right)=\frac{FP}{\left(FP+TN\right)}$$

## 4. Results

The obtained results for accuracy and loss, corresponding to each experimental protocol, along with the comparison of three feature selections are discussed here in five subsections, namely Section 4.1, Section 4.2, Section 4.3, Section 4.4 and Section 4.5, for accuracy vs. loss curves, results of experimental protocol 1, results of experimental protocol 2, results of experimental protocol 3, and comparison of three feature selections, respectively. The obtained results concur with our hypothesis, hence proving our hypothesis to be correct.

### 4.1. Accuracy and Loss Curves

**Figure 3:**
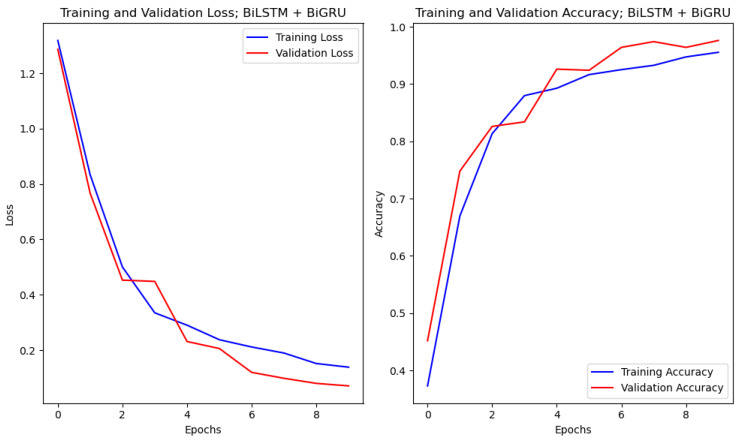
(**Left**) Loss vs. epochs plot for the BiLSTM + BiGRU model; (**Right**) accuracy vs. epochs plot for the BiLSTM + BiGRU model.

The model accuracy and the loss were plotted against the epochs used in the system. The accuracy of the model increased with the increasing number of epochs, whereas the loss decreased with the increasing epochs for both training and validation. The curve showed the exponential rise for the accuracy plot and the exponential decay for the loss functions. The loss function considered was categorical cross entropy. The accuracy and loss curve for the BiLSTM + BiGRU models are displayed in Figure 3 as an example of where the Adam optimizer is applied.

### 4.2. Results of Experimental Protocol 1

Table 1 details the results parameter for the unidirectional DL systems, namely RNN, GRU, and LSTM, for the different protocols (K2, K4, K5, and K10). The LSTM architectures have the highest accuracy and AUC in all the protocols. The K10 protocol provides the highest accuracy for all the unidirectional DL systems. The AUC ranges from 0.883 to 0.907 for all three models in the K2 protocol, 0.850 to 0.908 in the K4 protocol, 0.895 to 0.916 (RNN, GRU, and LSTM) in the K5 protocol, and 0.896 to 0.918 for all three models (RNN, GRU, and LSTM) in the K10 protocols.

### 4.3. Results of Experimental Protocol 2

The results obtained for the different bidirectional DL systems are displayed in Table 2. The different protocols used were K2, K4, K5, and K10. It can be seen that the BiLSTM architecture has the highest accuracy and AUC in all the protocols as well as, the best results in obtained in the K10 protocol for all the bidirectional DL models. The BiLSTM architecture has the ability for bidirectional mapping and enhanced contextual understanding that results in higher performance. The BiLSTM models provide flexible manipulation or exploration of the latent space.

### 4.4. Results of Experimental Protocol 3

EP3 featured the HDL systems. Table 3 details the different parameters that are obtained for the 12 combined HDL systems. The result showed that the AUC ranged from 0.930 to 0.974 in the K2 protocol, 0.931 to 0.975 in the K4 protocol, 0.947 to 0.985 in the K5 protocol, and 0.948 to 0.985 in the K10 protocol, showing the best performance in the K10 protocol. Among all the HDL systems, the combination of bidirectional DL systems (BiRNN + BiGRU, BiRNN + BiLSTM, and BiLSTM + BiGRU) results in the highest AUC.

### 4.5. Comparison of Three Feature Selection Methods for Three Machine Learning Models

The top important features were identified by using the three methods, namely PCA, CST, and RFR. The features selected are displayed in Table 4. The top ten commonly selected features are age, diabetes T1D, avg system before angio, IPN, creatinine, hyperlipidemia, and alpha-blockers. The mean accuracy increased with the involvement of these features. The mean accuracy of the HDL models is 0.956 with the selection of the features.

## 5. Performance Evaluation

The evaluation of any AI system must include the performance of the AI system. This includes the prediction and its comparison with gold standard labels, which can be accomplished by analyzing the receiver operating characteristics (ROC). Further, we are interested in understanding the effect of the training data size on the performance of the AI system. Lastly, we must quantify how HDL models meet the hypothesis that they are superior to unidirectional DL, bidirectional DL, and ML systems. In this context, the ROC is presented in Section 5.1, the effect of sample size is shown in Section 5.2, and the superiority of HDL models over other models is shown in Section 5.3.

### 5.1. Receiver Operating Characteristics of All Four Kinds of AI Models

**Figure 4:**
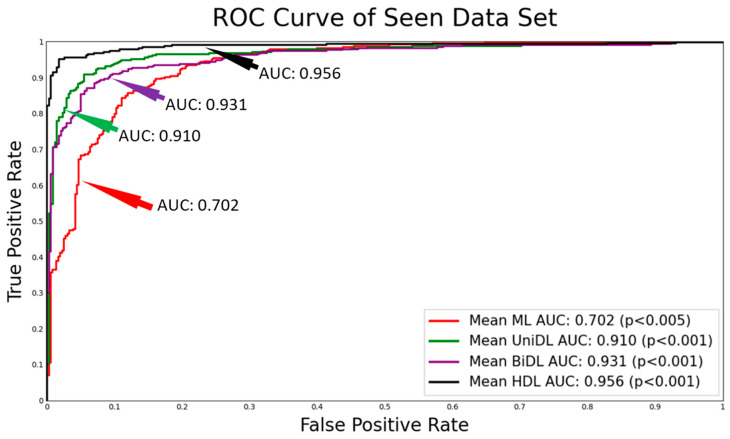
ROC showing the mean AUC along with their *p*-values; AUC: area-under-the-curve; ML: machine learning; DL: deep learning; UniDL: unidirectional DL; BiDL: bidirectional DL; HDL: hybrid DL.

Figure 4 shows the ROC curves obtained for all four clusters of the system, namely ML, unidirectional DL, bidirectional DL, and HDL, in increasing order of AUC values with corresponding *p*-values. The HDL cluster has the highest mean AUC value of 0.964 represented in the black color curve, whereas it can be observed that the ML system has the lowest mean AUC of 0.702 represented in the red color curve. In between these two curves lies the curve for the unidirectional DL in green color with a mean AUC of 0.910 and bidirectional DL in violet color with a mean AUC of 0.931. The fusion of unidirectional DL and bidirectional DL models demonstrates the rationale for the highest AUC validating our hypothesis. The corresponding *p*-values ensure the significance of the AUC values.

### 5.2. Effect of Sample Sizes on the Training System

The idea behind this experiment is to understand the effect of training data size on the performance of the AI models. This will assure us that the training models can better generalize, unlike memorization. To accomplish this experiment, we have taken four sets of paradigms, namely K2, K4, K5, and K10, which are computed for all the AI models. To appreciate the effect of data size on the training of the models, we compute the “stacked accuracy for (SA)” by stacking the accuracies for each of the cross-validation protocols.

**Figure 5:**
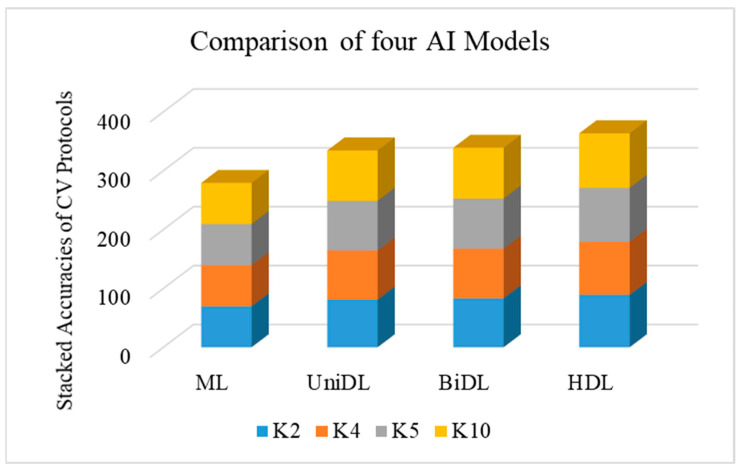
Plots for the effect of data size in the four model types; ML: machine learning; DL: deep learning; UniDL: unidirectional DL; BiDL: bidirectional DL; HDL: hybrid DL.

This stacking accuracy is computed for all four AI models. This allows for a powerful form of visual representation. Figure 5 shows the effect of training data size for all four models using the stacked accuracy concept. As seen in the figure, we can appreciate the color bands of each block gradually increasing compared to the previous color block. The best increase in color bands was seen for the HDL model, unlike the unidirectional, bidirectional, and machine-learning models. This validates our assumption that the performance improves with an increase in the training data size. This also validates that the increase in accuracy is very gradual and symptomatically settles down to nearly constant values even if the training set is 90% of the data size.

### 5.3. Hybrid Deep Learning Performance against Other AI Models

While HDL has shown superior performance compared to ML, unidirectional, and bidirectional models, it is important to quantify the absolute improvement in the difference between HDL and other AI models. The HDL system showed an improvement of 30.20% over the ML system using the seen datasets (since the mean accuracy of HDL was 90.88%, and the mean accuracy of ML was 69.80%; thus, the improvement was computed as the absolute difference of the mean accuracy of HDL minus the mean accuracy of ML divided by the mean accuracy of ML times 100, i.e., |90.88–69.80|/69.80 × 100~30.2%). Table 5 displays the absolute percentage increase improvement in HDL models when compared to ML, unidirectional DL, and bidirectional DL models. Our observations show that the HDL model is superior to ML, unidirectional DL, and bidirectional DL models by **30.20%**, **8.72%**, and **7.26%,** respectively.

## 6. Scientific Validation

**Figure 6:**
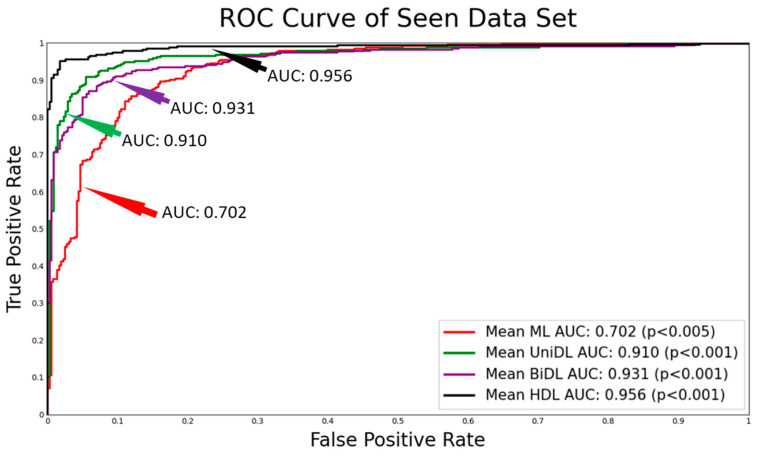

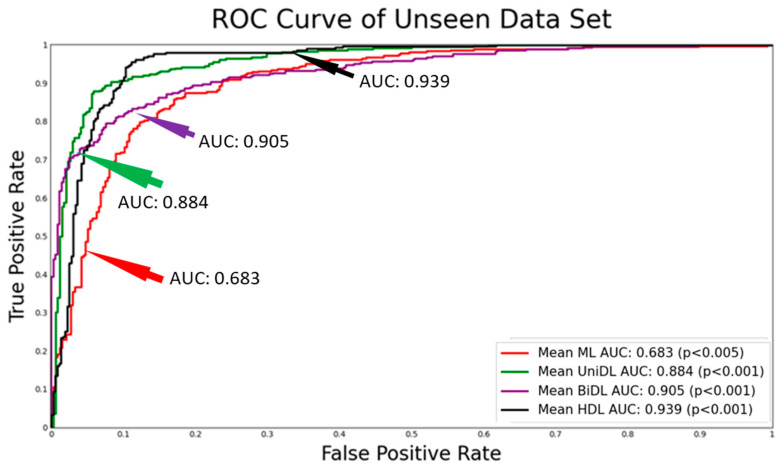
Receiver operating characteristic curve for mean AUC; (**Top**): seen dataset; (**Bottom**): unseen dataset.

The best way to validate the AI system is to evaluate the AI on a dataset that was never part of the original datasets [91,92]. This means the training was conducted on dataset A and testing on dataset B, where dataset B was never part of data A (See Appendix E, Table A2) [93]. To conduct the scientific validation, we took dataset A as the experimental dataset and dataset B as the validation dataset from another source having 20 risk predictors. Thus, our validation dataset consisted of 459 subjects with 20 risk features. Table 6 shows side by side the mean AUC values (along with their *p*-values) for experimental data (seen dataset) and validation data (unseen data). If one can show that the difference between these results has a 5% range, the AI model can be characterized for regulatory compliance. The percentages difference among the *seen* and *unseen* data were **2.78%**, **2.94%**, **2.87%**, and **1.79%** for ML, unidirectional DL, bidirectional DL, and HDL systems, respectively. We did observe that HDL models showed the lowest absolute difference between the experimental data (see analysis) and validation data (unseen data analysis) (see Table 6, Figure 6).

## 7. Reliability and Stability of Hybrid Deep Learning System

The reliability analysis was performed for the unidirectional (RNN) vs. bidirectional (BiRNN), HDL model (BiGRU + BiRNN) vs. unidirectional model (RNN), and HDL model (BiGRU + BiRNN) vs. bidirectional model (BiRNN), and more, with the K10 protocol by using statistical tests, namely the Mann–Whitney test. A non-parametric statistical test called the Mann–Whitney U test is used to see if there is a difference between two independent groups. It can be used for ranking, summing ranks, test statistics, and comparing critical values. It is specifically designed for paired data or repeated measures, where each subject or entity is measured twice under different conditions. The *p*-values were calculated and are displayed in Table 7. The *p*-values obtained are very similar, being <0.05 in all three compared models, hence validating that the systems are reliable and stable. A thorough selection of models utilizing empirical data is necessary, as indicated by the consistently low *p*-values, which show significant and persistent changes in prediction accuracy.

## 8. Discussion

The proposed novel study is unique in its application of HDL mechanisms for CVD risk stratification. Here, we have combined the angiographic scores (gold standard) with the risk variables such as CUSIP, IPN, and the conventional clinical risk factors. Leveraging the AtheroEdge™ 3.0_HDL_ architecture, this is the first study where the combination of traditional risk factors, laboratory-based factors, image phenotypes (radiomic features), and MedUSE risk factors was implemented in an HDL framework and benchmarked against machine learning, unidirectional DL, and bidirectional DL frameworks. The study demonstrated six HDL models. In combination A, we have three combinations, namely RNN + GRU, RNN + LSTM, and LSTM + GRU. In combination B, there are three combinations, namely BiRNN + BiGRU, BiRNN + BiLSTM, and BiLSTM + BiGRU. For input into the AtheroEdge™ 3.0_HDL_ architecture, the top 10 important features were identified via three methods, namely PCA, chi-square, and RFR. All three methods showed that 70% of features were shared amongst these three methods. The HDL system, AtheroEdge™ 3.0_HDL_, was more accurate for risk stratification of CVD than bidirectional DL, unidirectional DL, and ML models. Further, our study showed a ~30.20% improvement in the AUC for predicting the CVD in the HDL framework compared to the ML strategy. A statistical test, namely the Mann–Whitney test, was carried out to test the statistical reliability of the HDL AI system. For generalization of the AtheroEdge™ 3.0_HDL,_ the training was performed using experimental datasets, and testing was performed on the validation datasets. Such a system showed less than 1% difference between the seen data analysis and unseen data analysis, which also satisfies regulatory compliance, where the requirement is less than 5% [94,95].

### 8.1. Benchmarking Table

The benchmarking table for CVD risk stratification, which includes eight DL-based studies, is shown in Table 8. The characteristics include the author, the total features used (NOF), the strategies used, the total number of subjects/images, the cross-validation (CV) methodology, the survival analysis (SA), and the outcomes.

Nine risk variables were present in each of the 2406 patients in the Australian datasets utilized in the first analysis by Unnikrishnan et al. [96] (R1). They have chosen to use the conventional cardiovascular risk calculator (CCVRC) and linear regression classifiers in the K5 protocol for the Framingham Risk Score (FRS). The authors demonstrated that, in contrast to the ML-based calculators’ 0.71 AUC, the 10-year risk assessment’s AUC for CCVRC was 0.57. Next, an investigation by Jamthikar et al. [56] (R2) compared ML calculators, such as RF, GB, and SVM, with conventional calculators, such as the systematic coronary risk evaluation (SCORE), FRS, and extreme atherosclerotic cardiovascular disease (ASCVD). A total of 500 participants with 39 risk variables were used in this investigation. The authors demonstrated a significant difference in AUC between the CCVRC and ML models, with *p*-values less than 0.0001 and AUC values of 0.50 and 0.95, respectively. In the third study, which was conducted by Alaa et al. [19] (R3), the authors used a five-year follow-up technique to show how well ML classifiers performed versus the FRS algorithm for a UK dataset containing 423,604 subjects with 473 variables. As compared to CCVRC, the outcomes for ML classifiers were more accurate. The ML classifiers were AdaBoost, RF, SVM, and gradient-boosted machine (GBM) models with an AUC equal to 0.774 for the ML and 0.724 for the CCVRC.

The use of carotid imaging for CVD risk stratification has been increasing. Zhou et al., the fourth study (R4) [97], demonstrated the use of the UNet++ model for plaque segmentation in a multi-ethnic database. Depending on the CV methodology that was employed, the training and testing datasets included 100 and 44 images, respectively. Due to the small amount of data, the system was unable to be adequately justified for the hospital settings. They did not even benchmark the system against other cutting-edge systems. The HDL models (SegNetUNet and SegNetUNet+) were presented for segmentation of the plaque in a different work by Jain et al. [98] (R5). The internal carotid artery (ICA) was the section of the artery under consideration. To improve the datasets, the rotation transformation augmentation technique was used. The method’s shortcomings included racial bias in data selection and source identification. Two sets of multi-ethnic CCA datasets from Japan and Hong Kong were employed by Jain et al. [99] (R6) in their tests. To prevent various biases, they suggested using the HDL architecture for the unseen dataset. Nevertheless, the authors neglected to validate the suggested method against any existing CVD risk prediction systems on the market, which resulted in bias in the system’s validation. The seventh study taken into consideration was authored by Jain et al. [100] (R7), in which the solo DL (SDL) models and HDL models are contrasted with the different available systems in the market (AtheroEdgeTM 2.0) suggested by AtheroPointTM LLC, CA, USA. TPA inaccuracies for HDL (8 mm^2^), SDL (9.9 mm^2^), and traditional models (9.6 mm^2^) were displayed in the results for the image datasets. Johri et al. [54] (R8) show the use of 39 features with the ML models, namely RF and SVM. The used DL models were RNN and LSTM, applied to data from a total of 500 patients by following the K10 protocol. The results obtained were an AUC for the DL model of 0.99, for ML models 0.89, and for the CCVRC AUC 0.50. The last considered study by Akari et al. [48] (R9) uses 56 features for 4004 patients and only applied the SVM model. The protocol used is K10. The results show an accuracy of 98.43 and a reliability index of 97.32%.

Our system AtheroEdge™ 3.0_HDL_ uses HDL systems that include the coronary angiography (gold standard) and ultrasound (contrast-enhanced, carotid B-mode) as part of the risk variables. Our novel model consists of three ML models, three unidirectional DL models, three bidirectional DL models, and 12 DL-based hybrid models. The results showed that the bidirectional DL models are better performing when compared to the unidirectional DL. There was around a **30.20%** improvement in the HDL models over the previous ML-based systems for stratification of CVD risk in the same Canadian cohort. We have also performed statistical tests to prove the reliability of the HDL systems. Furthermore, the top 10 features were selected by using three methods. Lastly, the validation was performed with the unseen dataset and obtained a **~1%** difference in the results for the HDL systems.

### 8.2. A Special Note on Hybrid Deep Learning

Hybrid models increase overall performance in capturing complicated patterns by utilizing the advantages of many neural network designs. HDL models are adaptable to certain tasks, enabling the best elements to be chosen for each aspect of the issue. Transfer learning is made easier via the integration of pre-trained models, which allows for effective knowledge transfer between tasks and domains. HDL models function as ensembles, using several modelling techniques to minimize over fitting and improve resilience. They could provide improved interpretability since transparently constructed components make it easier to comprehend the judgments made via the model. HDL models may balance computing demands more effectively, which makes them appropriate for use in contexts with limited resources. HDL models use specific structures for each modality to manage multimodal data efficiently. Improvements in neural network research may be readily incorporated into HDL models, guaranteeing flexibility in response to new methods. Because of HDL models’ flexibility, task-specific architectures may be designed to maximize performance for certain goals. HDL designs, which increase model applicability, frequently result in higher generalization on a variety of datasets by merging many models.

### 8.3. Strengths, Weaknesses, and Extensions

The HDL-based system was initially put forth. Focused carotid B-mode ultrasound, contrast-enhanced ultrasonography, and coronary angiography were all used in the AtheroEdge 3.0_HDL_ planned system. The HDL-based models have deep layers that produce a generalized training model when trained with epochs, optimal batch sizes, and learning rates. When compared to ML outcomes, this yields better HDL results. Fusing LBBM and OBBM with radionics-based features provided by MedUSE, CUSIP, and IPN features is another benefit of AtheroEdge 3.0HDL. Three techniques were employed to choose the ML features to improve performance. The effect of different CV protocols was also studied for four types of AI models. The validation of the proposed HDL model was accomplished by applying it to unseen dataset which showed a difference of 1%. The Canadian ethnicity datasets were considered for both seen and unseen data.

The following are the system’s weaknesses and how they were mitigated: (i) *retraining of models*: the models need to be retrained in light of the altered input data. This modification might involve changing the sample size (the total number of patients) or adding new risk factors. There are ways to mitigate the effects even while retraining from stretch is necessary, such as by using transfer learning models; (ii) *sizes of training models*: AI models are always susceptible to having big training sets. Numerous factors are to blame for this. The AI techniques are under additional stress in terms of model sizes of the training patches due to the growth of risk factors and patient cohorts. Pruning AI algorithms is one mitigating strategy, notwithstanding the possibility of larger model sizes [101]; (iii) *AI bias*: due to a variety of factors, including bias in the prediction system, algorithm, and input datasets, bias in AI is a constant problem. Although bias can be present at many stages in the design of an AI system, there are mitigation techniques that allow us to identify and get rid of AI bias, including algorithms like Butterfly, PROBAST, and ROBINS methods [102]; (iv) *restrictions with hardware*: AI techniques that operate on the CPU clusters often require additional processing time. This depends on the quantity of data, batch sizes, learning rate, and epochs. GPU clusters allow for the mitigation of these variables, even if they have an impact on performance time [103]; (v) the proposed model applies only the CCE loss function [104,105,106]. Other loss function types can also be implemented. (vi) More performance comparisons can be conducted once the data sharing protocols are adopted.

In terms of improvements to the current study, distinct forms of plaque, such as symptomatic and asymptomatic plaque identified using the modality of grayscale type, could be included as one of the risks variable for CVD/stoke risk assessment [107,108,109]. Conventional approaches for wall segmentation can be used for CUSIP measurements [110,111,112]. In non-conventional approaches, sophisticated DL algorithms for CUSIP measurements can enhance the wall segmentation approaches [113,114,115]. The big data idea may be used in subsequent research to improve the accuracy of CVD risk forecasts [116,117,118,119,120]. To lower down the training sizes of the AI models, evolutionary approaches can also be used [47,101,121,122,123]. Some advanced models like (a) attention-based and (b) transformer-based models can be added to the underlying AI models [124,125,126], such as unidirectional and bidirectional models. While the above AI techniques utilize 2D ultrasound images, we can also extend this to 3D ultrasound [127,128,129]. *Additionally, we would like to attempt the combination of three models such as LSTM, RNN, and GRU* via *the nested fusion approach.*

## 9. Conclusions

Our technique is unique in that it employs HDL models with targeted carotid ultrasound (B-mode) as one of the risk predictors and contrast-enhanced ultrasonography with angiography of the coronary as the ground truth. Three machine learning models as well as three unidirectional, three bidirectional, and twelve hybrid models made up our system. Bidirectional models outperform unidirectional ones. Furthermore, using the same Canadian population, the HDL systems demonstrated a **30.20%** improvement over the prior ML-based method for CVD risk classification. Additionally, we have run the statistical tests necessary to demonstrate the HDL systems’ dependability. Additionally, three methodologies were used to choose the top ten attributes. Finally, the validation was carried out using an unknown dataset, and the findings for the HDL systems showed a difference of less than **1%**. The effect of data size was also studied showing the increase in the accuracy with the increasing K protocol. Further, different types of loss functions can be implemented in future work. The AtheroEdge 3.0_HDL_ system works in both an online and offline manner.

## Figures and Tables

**Table 1:** Results for all unidirectional DL models for all cross-validation protocols.

CVP	Models	ACC (%)	SPEC (%)	SEN (%)	*p*-Value	AUC (0–1)
K2	RNN	80.11	82.96	81.87	<0.001	0.883
	GRU	81.12	83.32	82.16	<0.001	0.892
	LSTM	81.81	85.67	83.36	<0.001	0.907
K4	RNN	82.52	81.97	83.88	<0.001	0.850
	GRU	83.13	82.33	84.17	<0.001	0.897
	LSTM	84.82	84.68	85.37	<0.001	0.908
K5	RNN	83.50	82.95	84.86	<0.001	0.895
	GRU	84.11	83.31	85.15	<0.001	0.900
	LSTM	85.80	85.66	86.35	<0.001	0.916
K10	RNN	84.20	83.15	83.86	<0.001	0.896
	GRU	85.12	84.11	86.15	<0.001	0.902
	LSTM	86.80	86.67	87.35	<0.001	0.918

CVP: Cross-Validation Protocol; ACC: Accuracy; SPEC: Specificity; SEN: Sensitivity; AUC: Area-Under-the-Curve.

**Table 2:** Results for all bidirectional DL models for all K protocols.

CVP.	Models	ACC (%)	SPEC (%)	SEN (%)	*p*-Value	AUC (0–1)
K2	BiRNN	82.22	80.16	82.34	<0.001	0.891
	BiGRU	83.42	81.17	83.18	<0.001	0.905
	BiLSTM	84.32	83.29	84.12	<0.001	0.913
K4	BiRNN	83.28	82.66	83.94	<0.001	0.910
	BiGRU	84.24	83.12	84.28	<0.001	0.915
	BiLSTM	85.13	85.68	86.52	<0.001	0.923
K5	BiRNN	84.28	83.66	84.94	<0.001	0.920
	BiGRU	85.24	84.12	85.28	<0.001	0.925
	BiLSTM	86.13	86.68	87.52	<0.001	0.933
K10	BiRNN	85.27	84.65	85.93	<0.001	0.920
	BiGRU	86.23	85.11	86.27	<0.001	0.925
	BiLSTM	87.00	86.67	88.51	<0.001	0.935

CVP: Cross-Validation Protocol; ACC: Accuracy; SPEC: Specificity; SEN: Sensitivity; AUC: Area-Under-the-Curve.

**Table 3:** Results for all HDL models for all K protocols.

CVP	Models	ACC (%)	SPEC (%)	SEN (%)	*p*-Value	AUC (0–1)
K2	RNN + GRU	85.00	82.76	82.62	<0.001	0.930
	RNN + LSTM	85.91	85.37	83.56	<0.001	0.939
	LSTM + GRU	88.02	88.16	84.62	<0.001	0.943
	BiRNN + BiGRU	90.53	89.75	88.32	<0.001	0.954
	BiRNN + BiLSTM	91.84	90.18	88.32	<0.001	0.968
	BiLSTM + BiGRU	94.14	92.22	89.12	<0.001	0.974
K4	RNN + GRU	86.66	83.76	83.62	<0.001	0.931
	RNN + LSTM	86.02	86.37	84.56	<0.001	0.940
	LSTM + GRU	89.02	89.16	85.62	<0.001	0.944
	BiRNN + BiGRU	91.53	90.75	87.32	<0.001	0.955
	BiRNN + BiLSTM	92.84	91.18	89.32	<0.001	0.969
	BiLSTM + BiGRU	95.14	93.22	90.12	<0.001	0.975
K5	RNN + GRU	86.45	82.76	84.62	<0.001	0.947
	RNN + LSTM	86.50	85.37	86.56	<0.001	0.957
	LSTM + GRU	91.02	88.16	87.62	<0.001	0.959
	BiRNN + BiGRU	93.53	84.75	87.32	<0.001	0.972
	BiRNN + BiLSTM	94.84	86.18	87.32	<0.001	0.976
	BiLSTM + BiGRU	96.14	92.22	93.12	<0.001	0.982
K10	RNN + GRU	88.14	82.75	84.61	<0.001	0.948
	RNN + LSTM	88.24	84.36	85.55	<0.001	0.958
	LSTM + GRU	92.01	87.15	86.61	<0.001	0.960
	BiRNN + BiGRU	94.52	88.74	87.31	<0.001	0.975
	BiRNN + BiLSTM	95.83	89.17	87.31	<0.001	0.979
	BiLSTM + BiGRU	97.25	92.21	93.11	<0.001	0.985

CVP: Cross-Validation Protocol; ACC: Accuracy; SPEC: Specificity; SEN: Sensitivity; AUC: Area-Under-the-Curve.

**Table 4:** The top ten selected features for all three methods.

SN	PCA	CST	RFR
1	Age	Age	Age
2	Diabetes T1D	Diabetes T1D	Diabetes T1 D
3	Avg Sys before angio	Avg Sys before angio	Avg Sys before angio
4	IPN	IPN	IPN
5	Creatinine	Creatinine	Creatinine
6	Hyperlipidemia	Hyperlipidemia	Hyperlipidemia
7	Alpha-Blockers	Alpha-Blockers	Alpha-Blockers
8	Insulin	Family Hx of CVD	Current Smoker
9	Angina	Current Smoker	BMI
10	Anti-Platelet/Anti-Coagulants	Anti-Platelet/Anti-Coagulants	TPA

SN: serial number; PCA: principal component analysis; CST: chi-square test; RFR: random forest regressor; IPN: intraplaque neovascularization; BMI: body mass index; TPA: total plaque area.

**Table 5:** Comparison of ML vs. unidirectional DL vs. bidirectional DL vs. HDL.

SN	M1 (a)	M2 (b)	% Increase (a − b/a) × 100
1	ML	HDL	30.20%
2	UniDL	HDL	8.72%
3	BiDL	HDL	7.26%

ML: machine learning; DL: deep learning; UniDL: unidirectional DL; BiDL: bidirectional DL; HDL: hybrid DL.

**Table 6:** Mean AUC values for seen vs. unseen data.

Seen Data				Unseen Data		
SN	Models	Mean AUC (0–1)	*p*-Value	Mean AUC (0–1)	*p*-Value	Difference (%)
1	ML	0.702	<0.005	0.683	<0.005	2.78%
2	UniDL	0.910	<0.001	0.884	<0.001	2.94%
3	BiDL	0.931	<0.001	0.905	<0.001	2.87%
4	HDL	0.956	<0.001	0.939	<0.001	1.79%

ML: machine learning; DL: deep learning; UniDL: unidirectional DL; BiDL: bidirectional DL; HDL: hybrid DL.

**Table 7:** Reliability and stability results for best unidirectional vs. bidirectional hybrid DL models.

SN	Model1	Model2	Mann-Whitney
1	BiRNN	RNN	*p* < 0.05
2	BiGRU + BiRNN	RNN	*p* < 0.05
3	BiRNN + BiLSTM	BiRNN	*p* < 0.05
4	BiRNN + BiLSTM	RNN	*p* < 0.05
5	BiRNN + BiGRU	BiRNN	*p* < 0.05
6	BiRNN + BiGRU	RNN	*p* < 0.05
7	BiLSTM + BiGRU	RNN	*p* < 0.05
8	BiLSTM + BiGRU	BiRNN	*p* < 0.05

**Table 8:** Benchmarking.

C0	C1	C2	C3	C4	C5	C6	C7
SN	Authors	NOF	ML/DL Models Used	#Patients/#Images	CV	SV	Results Obtained
R1	Unnikrishnan et al. [96]	09	SVM	2.4 K	K5	🗴	AUC for ML = 0.71; for CCVRC = 0.57
R2	Jamthikar et al. [56]	39	RF, SVM, XGBoost	500	K10	✓	AUC for ML = 0.95; for CCVRC = 0.50
R3	Alaa et al. [19]	473	SVM, GBM, RF, AdaBoost	423.6 K	K10	🗴	AUC for ML = 0.724; for CCVRC = 0.774
R4	Zhou et al. [97]	🗴	UNet++	144/510 497/638	K5	🗴	TPA error = 5.55 ± 4.34 mm^2^; DSC = 83.3–85.7%
R5	Jain et al. [98]	🗴	UNet+, UNet, SegNetUNet, SegNet, Unet + SegNet	97/970	K5	🗴	AUC for UNet = 0.91, for UNet + = 0.91, for SegNet-UNet = 0.908, for SegNet = 0.905, and for SegNetUNet+ = 0.898 (using CE-loss models) and 0.883, 0.889, 0.905, 0.889, and 0.907 (using DSC-loss models); PA error = 3.49 mm^2^ for SDL and 4.21 mm^2^ for HDL
R6	Jain et al. [99]	24	UNet	379, 300	K10	🗴	Unseen FoM: 70.96 and 91.14 Seen FoM: 97.57, 88.89, and 99.14
R7	Jain et al. [100]	24	🗴 AtheroEdge 2.0, UNet, UNetSegNet	379	K10	🗴	AUC for UNet = 0.93, for SegNet-UNet = 0.94; for AtheroEdge™ 2.0 = 0.95, respectively; SDL PA error = 9.9 mm^2^; HDL PA error = 8 mm^2^; AtheroEdge™ = 2.0 mm^2^; PA error = 9.6 mm^2^
R8	Johri et al. [54]	39	RF, SVM, RNN, LSTM	500	K10	✓	AUC for DL AUC = 0.99, for ML = 0.89, for CCVRC = 0.50
R9	Akari et al. [48]	56	SVM	4004	K10	✓	ACC = 98.43; reliability index = 97.32%
R10	Proposed method	39	SVM, RF, XGBoost, UniDL, BiDL, HDL	500	K2, K4, K5, K10	✓	AUC for ML = 0.702, for UniDL = 0.910, for BiDL = 0.931, and for HDL = 0.956

SN: Serial number; NoF: #features; DL: Deep learning; ML: Machine learning; RF: Random forest model; SVM: Support vector machine model; GBM: Gradient boost machine model; CV: Cross-validation; SV: Scientific validation; RNN: Recurrent neural network; CCVRC: Conventional cardiovascular risk calculator; LSTM: Long short-term memory; AUC: Area-under-the-curve; SDL: Solo DL; TPA: Total plaque area; PA: Plaque area; HDL: Hybrid DL; ACC: Accuracy; uniDL: Unidirectional DL; BiDL: Bidirectional DL; HDL: Hybrid DL.

**Figure A8:**
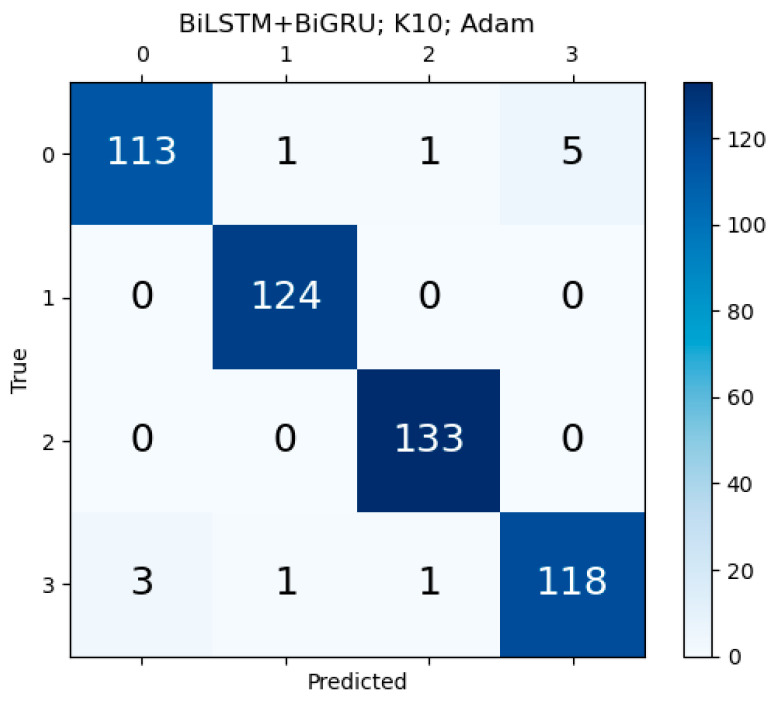
Confusion matrix for the best K10 protocol which fuses BiLSTM and BiGRU.

**Figure A9:**
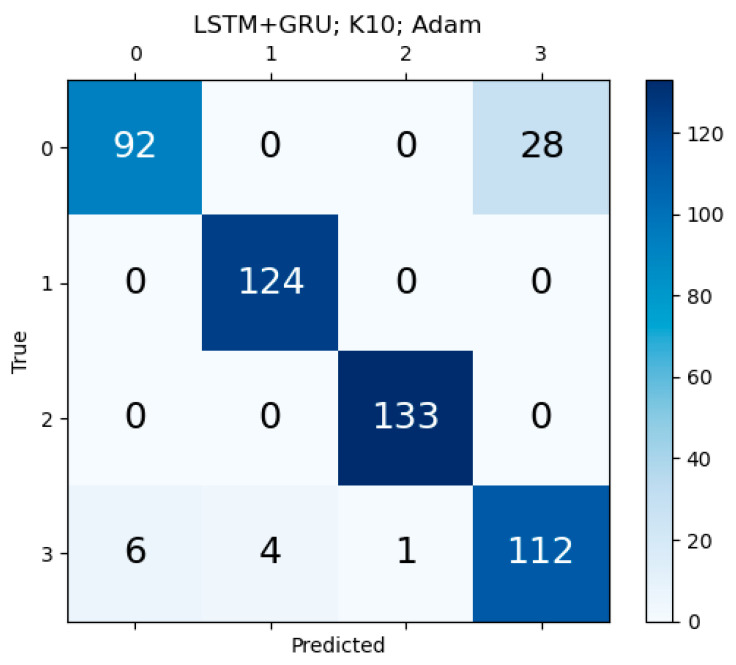
Confusion matrix for the best K10 protocol which fuses LSTM and GRU.

**Figure A10:**
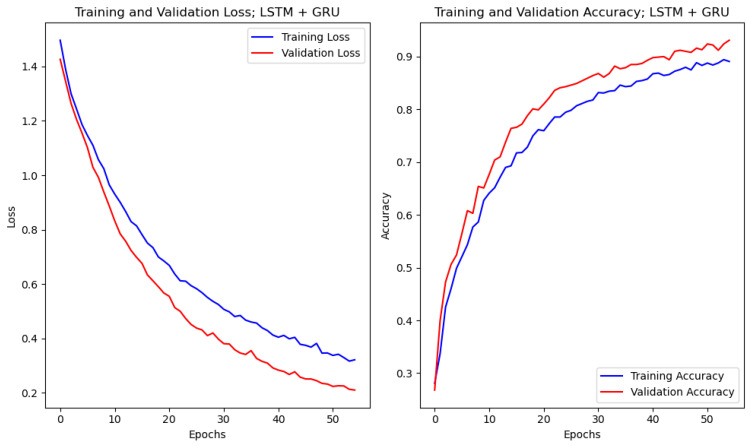
Loss and accuracy curves for the best K10 protocol which fuses LSTM and GRU.

## Data Availability

Data are not available due to the proprietary nature of this study.

## Appendix A. Unidirectional Architecture

**Table A1:** Baseline Characteristics.

SN	Risk Factors or Target Variable	Samples (N) or Mean	Pr (%)
1	Age	64.49 years	-
2	Gender	349	69.8
3	Obesity	215	43.0
4	Ethnicity	486	97.2
5	BMI	31.12 kg/m^2^	-
6	Hypertension	338	67.6
7	Angina	124	24.8
8	Diastolic blood pressure	76.7 mmHg	-
9	Systolic blood pressure	135.35 mmHg	-
10	Smoking history	330	66.0
11	Casual smoker	15	3.0
12	Current smoker	100	20.0
13	Previous smoker	218	43.6
14	Drinks	4.94 ± 10.4 per week	-
15	Family history of diabetics	195	39.0
16	Premature CVD in the family	146	29.2
17	CVD in the family	321	64.2
18	Pre-diabetic	20	40.0
19	Hyperlipidemia	288	57.6
20	Type II diabetes	114	22.8
21	Type I diabetes	5	1.0
22	Creatinine	83.99 ± 22.6 μmol/L	-
23	Estimated glomerular filtration rate	78.96 mL/min/1.73 m^2^	-
24	Diabetes of any type	118	23.6
25	Total plaque area (TPA)	47.68 mm^2^	-
26	Maximum plaque height (MPH)	2.64 mm	-
27	Intra-plaque neovascularization (IPN)	1.16	-
28	Angiotensin-converting enzyme (ACE) inhibitors	191	38.2
29	HMG-Co reductase inhibitors	272	54.4
30	Angiotensin receptor blockers (ARBs)	45	9.0
31	Other antilipemic agents	9	1.80
32	Alpha-blockers	30	6.0
33	Calcium channel-blockers	93	18.6
34	Beta-blockers	236	47.2
35	Diuretics	99	19.8
36	Anti-platelet	368	73.6
37	Insulin	38	7.6
38	Anti-anginals and NSAIDS	81	16.20
39	Non-insulin diabetes medications	72	14.4

## Appendix E. Scientific Validation with the Unseen Data

**Table A2:** Scientific validation with the unseen data.

Unseen Data (Train A-Test B)				Unseen Data (Train B-Test A)	
SN	Models	Mean AUC	*p*-Value	Mean AUC	*p*-Value
1	ML	0.683	<0.005	0.683	<0.005
2	UniDL	0.884	<0.001	0.880	<0.001
3	BiDL	0.905	<0.001	0.900	<0.001
4	HDL	0.947	<0.001	0.940	<0.001

SN: Serial Number; ML: Machine Learning; DL: Deep Learning; UniDL: Unidirectional DL; BiDL: Bidirectional DL; AUC: Area-Under-the-Curve; HDL: Hybrid DL.
